# How Cells Integrate Complex Stimuli: The Effect of Feedback from Phosphoinositides and Cell Shape on Cell Polarization and Motility

**DOI:** 10.1371/journal.pcbi.1002402

**Published:** 2012-03-01

**Authors:** Athanasius F. M. Marée, Verônica A. Grieneisen, Leah Edelstein-Keshet

**Affiliations:** 1 Computational & Systems Biology, John Innes Centre, Norwich Research Park, Norwich, United Kingdom; 2 Institute of Applied Mathematics and Department of Mathematics, University of British Columbia, Vancouver, British Columbia, Canada; University of California San Diego, United States of America

## Abstract

To regulate shape changes, motility and chemotaxis in eukaryotic cells, signal transduction pathways channel extracellular stimuli to the reorganization of the actin cytoskeleton. The complexity of such networks makes it difficult to understand the roles of individual components, let alone their interactions and multiple feedbacks within a given layer and between layers of signalling. Even more challenging is the question of if and how the shape of the cell affects and is affected by this internal spatiotemporal reorganization. Here we build on our previous 2D cell motility model where signalling from the Rho family GTPases (Cdc42, Rac, and Rho) was shown to organize the cell polarization, actin reorganization, shape change, and motility in simple gradients. We extend this work in two ways: First, we investigate the effects of the feedback between the phosphoinositides (PIs) 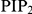, 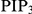 and Rho family GTPases. We show how that feedback increases heights and breadths of zones of Cdc42 activity, facilitating global communication between competing cell “fronts”. This hastens the commitment to a single lamellipodium initiated in response to multiple, complex, or rapidly changing stimuli. Second, we show how cell shape feeds back on internal distribution of GTPases. Constraints on chemical isocline curvature imposed by boundary conditions results in the fact that dynamic cell shape leads to faster biochemical redistribution when the cell is repolarized. Cells with frozen cytoskeleton, and static shapes, consequently respond more slowly to reorienting stimuli than cells with dynamic shape changes, the degree of the shape-induced effects being proportional to the extent of cell deformation. We explain these concepts in the context of several *in silico* experiments using our 2D computational cell model.

## Author Summary

Single cells, such as amoeba and white blood cells, change shape and move in response to environmental stimuli. Their behaviour is a consequence of the intracellular properties balanced by external forces. The internal regulation is modulated by several proteins that interact with one another and with membrane lipids. We examine, through *in silico* experiments using a computational model of a moving cell, the interactions of an important class of such proteins (Rho GTPases) and lipids (phosphoinositides, PIs), their spatial redistribution, and how they affect and are affected by cell shape. Certain GTPases promote the assembly of the actin cytoskeleton. This then leads to the formation of a cell protrusion, the leading edge. The feedback between PIs and GTPases facilitates global communication across the cell, ensuring that multiple, complex, or rapidly changing stimuli can be resolved into a single decision for positioning the leading edge. Interestingly, the cell shape itself affects the intracellular biochemistry, resulting from interactions between the curvature of the chemical fronts and the cell edge. Cells with static shapes consequently respond more slowly to reorienting stimuli than cells with dynamic shape changes. This potential to respond more rapidly to external stimuli depends on the degree of cellular shape deformation.

## Introduction

Reorganization of the actin cytoskeleton is essential in eukaryotic cell motility. Signalling modules that regulate this reorganization include the Rho GTPases (Cdc42, Rac, Rho) and membrane lipids (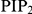 and 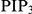). When a cell is stimulated by a graded or localized external signal, these internal signalling components redistribute on the timescale of seconds. Their redistribution defines the cell's polarization, determining the locations of the “front” and “rear” of the cell. In zones of high Cdc42 or Rac, actin filament barbed ends proliferate by Arp2/3-mediated branching [1]–[4], extend until they reach the membrane, and then exert internal forces against the membrane. In zones of high Rho activity, actomyosin contraction is enhanced [5]–[7]. These combined effects lead to protrusion at the cell front and retraction at the rear. Collectively, such effects change the cell's shape, and orchestrate directed motion and chemotaxis. How these pathways are coordinated in space and time, and how they affect/are affected by feedbacks with the dynamic cell shape are fundamental questions in the field. Recent work on visualizing cell motility *in vivo*, e.g. Yoo et al. [8], also points to the importance of understanding the role of feedback (e.g. between 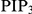 and Rac). This paper addresses such questions in the context of a computational model for cell motility.

**Figure 1:**
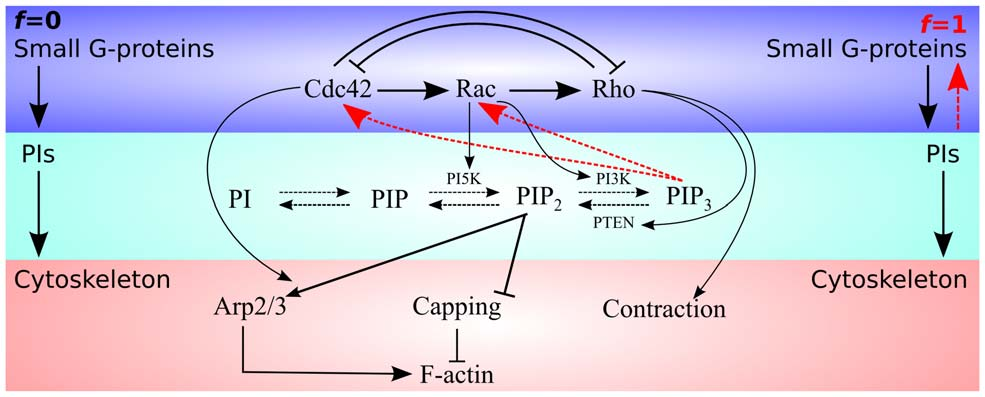
Signalling pathways assumed in the model. The top row represents small GTPases, the middle row depicts phosphoinositides, and at the lowest level are the cytoskeletal components. Here we explore the effects of feedback from the PIs to the small GTPases, indicated by the red dashed lines. The parameter 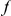 in the model represents the magnitude of the feedback (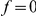 means the feedback is absent, 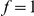 means it is essential for activating Cdc42 and Rac).

The Rho GTPases are switch-like proteins that cycle between active membrane-bound (GTP) forms and inactive cytosolic (GDP) forms. Activation is mediated by guanine exchange factors (GEFs), inactivation by GTPase activating proteins (GAPs), and extraction from membrane to cytosol is regulated by GDP dissociation inhibitors (GDIs). Rho family GTPases are universally found in eukaryotes, and highly conserved in evolution. Cdc42 and Rac signal to the actin nucleating complex Arp2/3, which in turn promotes actin branching, creation of new actin plus ends and local protrusion of the cell membrane [1]–[4]. Hence, regions in a cell where Cdc42/Rac activity are high tend to take on the role of a protrusive front. The small GTPase Rho activates actomyosin contractility, leading to local contraction [5]–[7] in regions of a cell that become the “rear”. The membrane lipids PI, PIP, 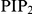 and 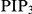, known as phosphoinositides (PIs), participate in signalling to the cytoskeleton. 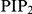 locally inhibits capping of actin filament ends and synergizes with Cdc42 in activating Arp2/3 [3]. The kinase PI3K (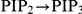) becomes upregulated at the location of a stimulated cell closest to an attractant pulse, whereas the phosphatase PTEN (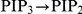) shifts to the opposite end. This, in turn, elevates 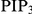 at what becomes the cell front [9]–[11]. There are multiple feedbacks in the signalling system. In neutrophils, there is evidence for mutual exclusion of Cdc42 and Rho [12]–[14]. Feedback between Rac and PI5K [15], [16] and PI3K [8], [17]–[19], feedback between 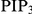 and Cdc42 and/or Rac [20]–[23], as well as feedback from Rho to PTEN [24] has been observed. Such feedbacks and interactions are depicted in Fig. 1, as previously discussed in Dawes and Edelstein-Keshet [25].

The roles of phosphoinositides and their kinase PI3K have come under renewed scrutiny in recent years. Based on experiments with the amoeba *Dictyostelium discoideum*, it was originally held that phosphoinositides (PIs) act as the “compass” that dictates the direction-sensing and chemotactic ability of cells [9], [26]–[29]. Indeed, if PI3K is inhibited by various treatments, cell polarity and cell motility are affected [29], mainly in shallow external gradients [30]. Recent evidence shows that inhibiting PI3K in neutrophils *in vivo* inhibits cell motility [8]. However, inactivating all genes that code for PI3Ks [31] or inhibiting PI3K with chemical treatment [30] in *Dictyostelium* does not destroy chemotaxis. Consequently, it is no longer clear what are the roles of the phosphoinositides in chemotaxis [32], [33]. This question motivates our investigation into the role of this signalling layer and its feedbacks. We explore how such feedback modulates and facilitates communication between regions of high GTPases activity, where such long-range communication is otherwise too slow. We point to aberrant behaviour that results when feedback is either absent or too strong.

A second theme in our paper is the effect of cell shape on the dynamics of signalling. Up to now, it has been well established that signalling cascades and their downstream effects can modulate and change the shape of a cell, causing protrusion, retraction, turning, reversal, and so on. However, whether cell shape and geometry also feeds back on signalling is less well-explored. Here we will show that the dynamic shape of a motile cell has downstream effects on intracellular protein patterning via a geometric effect that accelerates the repolarization of the cell in response to a new stimuli. We also discuss how PIs can influence shape-induced polarity behaviour.

### Modelling background

In order to address these issues, we have developed a computational model that integrates the signalling biochemistry with actin-based motility in a spatial setting. Our main philosophy in constructing the model has been to assemble modules of the signalling repertoire for which there is biological consensus or strong experimental evidence, to identify model parameters based on quantitative biological information, and to study the dynamics of these modules individually [34], [35], with dynamic actin cytoskeleton [25], [36] and in concert with other signalling modules [25] in 2D spatiotemporal computations. Details of the assumptions, steps, parameter choices and strategy have been extensively reviewed elsewhere [25], [34], [36] and are abbreviated in the Materials and Methods.

In view of our main aim to understand the role of feedback from PIs to GTPases, we here revised the model in Dawes and Edelstein-Keshet [25] so as to “tune” the magnitude of feedback from the PIs to the Rho GTPases over a full range (from absent to essential) and compare the resulting behaviours. (See dashed lines in Fig. 1). This *in silico* tuning represents our depiction of *in vitro* or *in vivo* knockout, silencing, and overexpression experiments. To meet our second aim of elucidating how cell shape influences biochemical repolarization, we implement a fully 2D computation with evolving cell geometry.

The biochemistry is summarized schematically in Fig. 1. The model consists of a set of coupled partial differential equations (PDEs) that describe the kinetics, crosstalk, diffusion, and exchange of the following intermediates: Cdc42, Rac, and Rho (active and inactive forms – 6 PDEs), PIP, 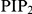, 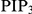 (lipids diffusing in the membrane – 3 PDEs) and Arp2/3 (active cytosolic form, 1 PDE). (See equations in the Materials and Methods and parameter values based on biological data in Table 1). Initially, all concentrations are uniform in the interior of a circular domain, representing an unstimulated resting cell. Stimulation is depicted by imposing a transient, spatially dependent activation of Cdc42 on a cell that is initially at rest, with no other spatial bias in any internal component. To keep the size of the model modest, we do not explicitly model the Rho GEFs and GAPs nor the kinases or phosphatases (PI5K, PI3K, PTEN) as dynamic variables. Feedback from GTPases to kinases and phosphatases are included in concentration-dependent rate-constants. In order to appraise the effect of PI feedback onto the small GTPases, we introduced a parameter, 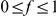 for the efficacy of such feedback (where 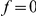 means no feedback from PIs and 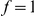 means that PIs are essential for activation of the small GTPases (see Eqn. (4)).

**Table 1:** Parameter estimates relevant to (i) actin dynamics; (ii) Rho-proteins; (iii) phosphoinositide dynamics; and (iv) cell surface mechanics, with sources from which they were obtained.

Parameter	Definition	Value	Source
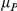	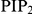 -dependent Arp2/3 activation rate	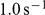	[25]
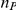	Hill coeff. of 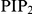 -mediated Arp2/3 activation	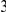	[25]
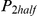	Threshold conc. of 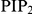 for Arp2/3 activation	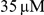	[25]
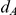	Arp2/3 decay rate	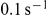	[36]
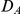	Arp2/3 diffusion coefficient	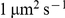	[93]
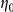	Arp2/3 nucleation rate	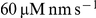	[36]
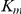	saturation constant for Arp2/3 nucleation	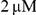	[36]
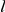	scale factor (converts units of 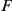 to conc.)	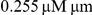	[36]
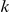	scale factor (converts conc. to units of 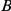 )	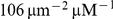	[36]
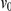	actin filament growth rate (free polymerization)	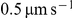	[94], [95]
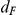	actin filament turnover rate	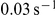	[88], [96]
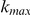	barbed end capping rate	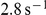	[95], [97]
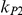	max reduction of capping by 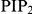	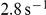	[25]
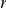	reduction in capping rate near leading edge	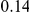	[36]
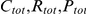	total levels of Cdc42, Rac, Rho	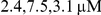	[36], [91], [98]
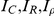	Cdc42, Rac, Rho activation input rates	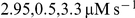	[36], [91]
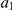	Rho level for half-max inhibition of Cdc42	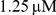	[36]
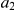	Cdc42 level for half-max inhibition of Rho	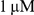	[36]
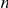	Hill coeff. of Cdc42-Rho mutual inhibition	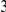	[36]
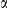	Cdc42-dependent Rac activation rate	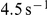	[36]
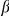	Rac-dependent Rho activation rate	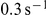	[36]
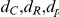	decay rates of activated Rho-proteins	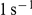	[99], [100]
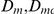	diffusion coeff. of active, inactive Rho-proteins	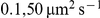	[62], [101]
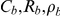	typical basal levels of active Cdc42, Rac, Rho	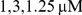	[36]
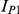	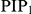 input rate	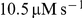	[92]
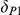	PIP 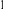 decay rate	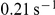	[92]
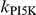	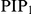 to 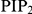 baseline conversion rate (by PI5K)	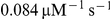	[92]
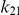	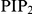 to 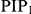 conversion rate	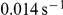	[92]
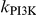	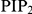 to 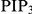 baseline conversion rate (by PI3K)	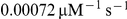	[92]
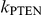	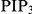 to 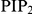 baseline conversion rate (by PTEN)	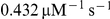	[92]
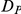	PI diffusion rate	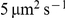	[101], [102]
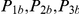	typical basal levels of 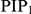 , 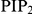 , 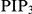	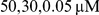	[70], [103]
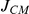	coupling energy per boundary site	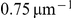	[36]
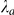	cell inelasticity	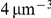	[36]
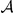	target area of the cell	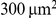	[36]
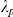	membrane inelasticity	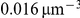	––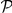	target perimeter of the cell	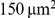	––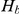	membrane yield	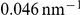	[36]
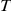	simulation “temperature”	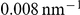	[36]
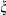	effect of Rho on contraction	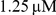	[36]
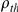	Rho contraction threshold	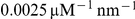	[36]

Note citations of our earlier works (i.e. [25], [36], [91]–[93]), where many detailed derivations, explanations, and references can be found.

As described in [36], the discretized densities and orientations of actin filaments and barbed ends are represented as evolving spatial distributions, where Arp2/3-dependent branching enhanced by Cdc42 nucleates new barbed ends. We keep track of a special category of barbed ends engaged with and applying force to the membrane, the “pushing barbed ends”. These promote local outwards protrusion as in the thermal polymerization ratchet mechanism [37]–[39]. Areas of high Rho are interpreted as sites where actomyosin contraction would be enhanced. This is depicted by a force directed inwards and perpendicular to the cell membrane. (Such zones tend to spontaneously become the “back” of the evolving cell.)

To combine reaction-diffusion (RD) kinetics with fully dynamic cell shape so as to show the important feedbacks between the geometry and the biochemistry, we use the Cellular Potts Model (CPM) framework [36], [40], [41]. In this multi-scale approach, the CPM specifies the domain and boundary conditions for the RD equations (PDEs) at each time point. The PDEs are solved efficiently “on the fly” in the irregular domain, generating the intracellular patterns that lead to differential forces on the cell membrane. The shape is then updated by an energy-minimization (Hamiltonian based) stochastic edge update algorithm [40], [41]. (See Materials and Methods.)

## Results

### Basic motility phenotype

**Figure 2:**
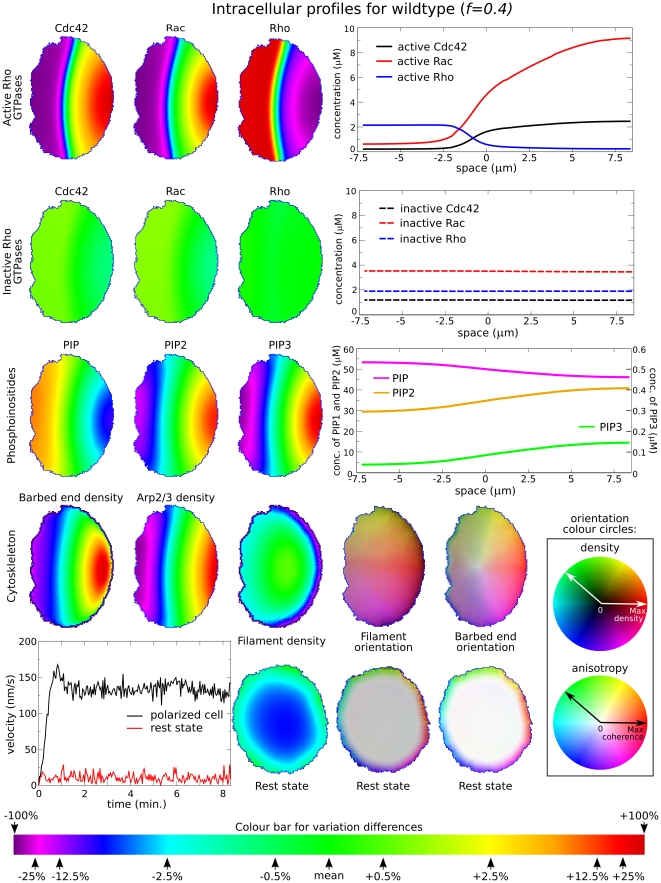
Basic motility phenotype produced by the “wildtype” model cell. The cell is initiated at rest, stimulated for 10 sec with a 15% gradient in the Cdc42 basal activation rates, and simulated for a total of 8 minutes, after which the stimulus is removed. (See Materials and Methods for details.) The figure presents a snapshot of the distributions and profiles when the cell has reached an effective steady state, i.e. when the cell shape and profiles do not further change, except for small fluctuations due to the stochastic nature of the formalism. The following is shown in the panels from left to right. Top row: intracellular steady-state distributions of active Rho GTPases Cdc42, Rac, and Rho, for the polarized cell state, followed by a graph of the steady-state profiles along the front-back axis of the cell; Second row: inactive Rho GTPases, and corresponding profiles; Third row: phosphoinositides PI, PIP2, PIP3 and corresponding profiles. Fourth row: barbed end density, Arp2/3, F-actin density, filament orientation and barbed end orientation distributions. Fifth row: left graph shows the velocity over time of a polarized (black) and a resting cell (red). The rest state, which is the second possible steady state in this system, is stable against low-amplitude noise. The corresponding distributions of filament density, barbed end and actin filament orientation for the resting cell are shown at the right. Colour map: (box) Cytoskeleton orientation is encoded using a colour-wheel where each orientation is represented by a colour at the given angle along the circle (hue). The filament density ranges from black (no filaments) to maximal density at the greatest colour intensity; distribution of filament orientations varies in saturation from white (complete anisotropy) to the maximal colour intensity (total coherence). (lower colour bar) Scale used for relative concentration/density “heat maps” in this figure. Note that the steepness of the internal gradient of signalling chemicals is reflected in the tightness of the transition between hues. The “front-back” interface is here taken as the isocline shown in green (labelled “mean”). Green is used to represent the mean rest state concentration value for any of the PIs, small GTPases, and Arp2/3. Deviations from this mean are captured by the heat map, in which the percentage variations above or below the mean, are as indicated by the arrows along this bar. (See also Video S1.)

To assess whether the model can capture basic experimental observations we ran the full model (Eqs. (1)–(16)) with biologically-based parameter values (Table 1). In the absence of stimuli, the resting cell is stable, and does not deform significantly nor move. We imposed a transient (10 s) gradient in the Cdc42 activation rate on the resting cell, and followed the dynamics of the GTPases (Eqs. 1, 2), the PIs (Eqs. 5), Arp2/3 (Eqn. 11), and actin (Eqs. 7–8) for 90 simulated minutes. Other than the graded stimulus, we do not *a priori* define a “front” or a “back” in the cell; all other dynamics develop spontaneously. The final cell shape and spatial distributions of all variables are shown in Fig. 2.

Profiles of the GTPases and PIs (rows 1–3) are shown both as 2D heat maps (left) and as line plots (right). As observed experimentally, active Cdc42 and Rac, as well as 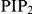 and 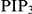, are enriched at one end, whereas active Rho and PIP are most prevalent at the opposite end. 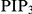 forms the steepest gradient, followed by 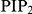. Due to their very rapid rates of diffusion in the cytosol, the inactive GTPases distribute more or less uniformly over the cell (Fig. 2, Row 2), even when their active forms are spatially segregated.

The transition between resting and motile cell is indicated in several panels in Fig. 2. In the rest state, the cell is disk shaped, with radially symmetric filaments and barbed end densities. Stochastic noise leads to a fluctuating edge and small displacements of the centre of mass, but the cell as a whole does not move (red curve, bottom left panel, Fig. 2). Once stimulated, the cell rapidly takes on a roughly oval shape and attains a velocity of 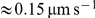. This speed remains constant and is maintained after the transient stimulus is removed, unless other stimuli are introduced (See Video S1, and black curve, bottom left panel, Fig. 2).

Elevated Rac and Cdc42 enhance Arp2/3 activation and branching of actin filaments. (Eqs. 7–8 and Fig. 2, Row 4.) In the case of Rac, this takes place through the activation of PI5K (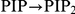), which elevates 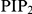 and in turn induces Arp2/3 activation. Cdc42 further accelerates the 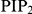-induced Arp2/3 activation, which promotes a local increase in barbed ends (Eqn. 8). The orientations and degree of anisotropy of the filaments and their barbed ends are indicated for the motile cell (Row 4) and resting cell (Row 5). Some barbed ends (Eqn. 13) contribute to a protrusive force that pushes out that part of the cell. This results in the spontaneous formation of a leading edge that defines the front of the cell. As seen in Fig. 2, Rho is highest at the rear of the polarized cell. This leads to a distributed isotropic inwards contractility that causes retraction, and formation of a trailing edge that becomes the “rear” of the cell.

Following a stimulus, there is a transient reorganization in the biochemistry and then a sharp transition is formed separating the zone of high Cdc42 activity (“front”) from the zone of low Cdc42 activity (“back”). We refer to the border between these zones as the “front-back interface”. For visual convenience, we use green to denote the mean concentration in all 2D chemical distributions, so the green isocline (see Cdc42, Row 1 Fig. 2) can be identified with that “front-back interface”. The appearance of robust polarization with a sharp transition zone recapitulates results of our previous models on GTPases [34]-[36], where we showed that the proximity of bistable kinetics, mass conservation, and disparity in the rates of diffusion of active and inactive GTPases leads to formation of a zero speed interface separating “front” and “back” in the cell (*wave-pinning*). (Even though with PI feedback the transition from front to back gets smeared out, in both cases we can select one of the intermediate isoclines to represent the demarcation between the front and the rear of the cell, and we informally refer to that boundary as an “interface” in both cases.)

As a cell edge extends outwards, chemical isoclines also relocate. Once the cell starts to move, it increases the region of “frontness”. But then, the buildup of active GTPase at the front is at the expense of the inactive GTPase pool. This means that the front-back interface moves forward, compensating for that depletion. A balance occurs when the speed of the front-back interface matches the forward motion of the leading edge of the cell, i.e. moves in perfect pace with the cell edge (see Video S1). In this sense, the system exhibits a self-correcting internal structure. We later discuss how perturbing this internal chemical distribution causes it to return to the basic robust polarization here described.

In view of the above, the basic model reproduces essential aspects of cell motility and reasonable distributions of the signalling chemicals and the cytoskeleton. We can now use this basic simulation run as a control against which to appraise *in silico* experiments.

### Effect of PIs on GTPase profiles and communication

**Figure 3:**
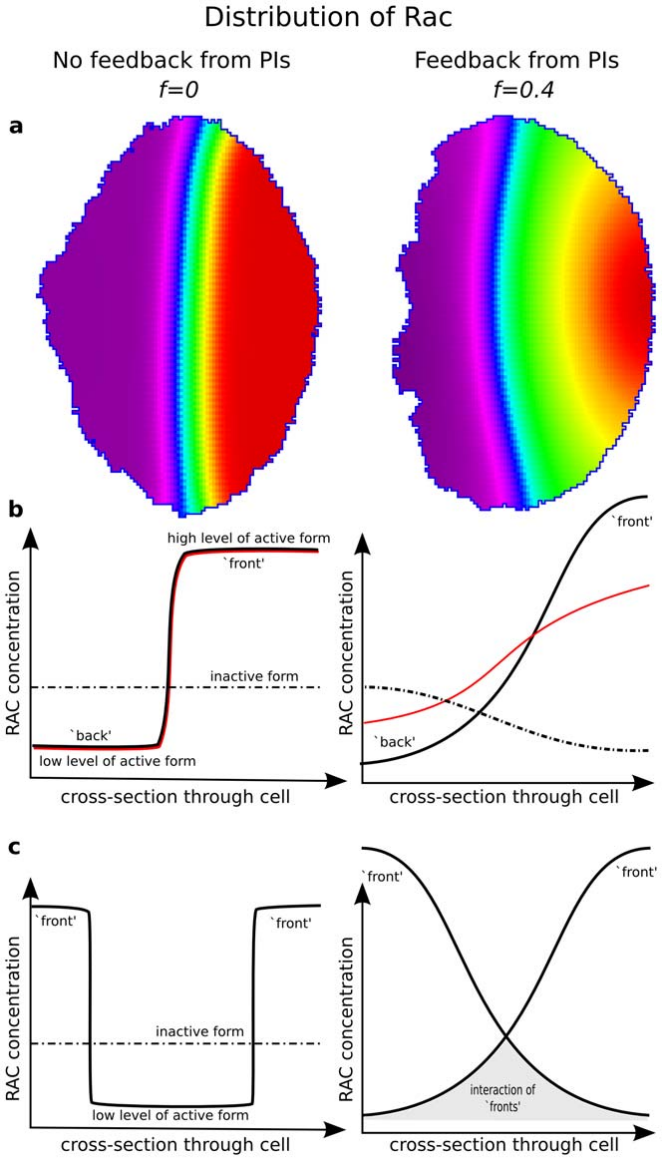
Schematics of how feedback from PIs change small GTPase profiles. Shown are the Rac distributions in the 2D cell [(**a**) and Video S1] and in a representative 1D cross-section along the cell diameter (**b,c**). Left panels: absent PI feedback (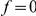). Right panels: with PI feedback (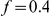). The sharp transition between high and low Rac activity is seen on the left (contours closely spaced), whereas PIs create a broader transition zone (right). Panels (b–c): schematics of differences in intracellular patterns due to maximal PI feedback to Rho proteins. (b): Inactive Rac (dot-dashed line) is nearly uniform for 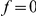, but shows significant depletion close to the “front” for high 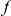. Decreasing the rate of diffusion 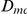 of inactive Rac (red curves) has little effect on the profile when 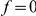. In contrast, decreasing 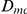 when 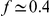 leads to a lower peak of active Rac at the front. Panels (c): Communication of multiple peaks of active Rac is very slow in the 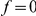 case, and much more significant in the case 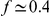. Hence, feedback from PIs helps to resolve conflicting cell “fronts”.

In Fig. 3 we contrast the GTPase profiles that occur with and without PI feedback in simulations (a), and schematically (b,c). When there is no PI feedback (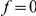) in this model, and in previous models where PIs were not explicitly included [34], [36], profiles of active GTPases are plateaus (high and low) separated by a narrow transition zone (a generic property, discussed mathematically in [35]). Without PI feedback, since active zones are flat plateaus, their interactions drop off steeply so that two such plateaus (Fig. 3c, left) hardly influence each other at a distance. Moreover, in this case, inactive GTPases (dot-dash line, Fig. 3b,c left) are essentially uniform in space [34], [36]. Increase or decrease in the total amount of the GTPase or its basic activation rate then determines the width of the region of activity.

Including PI feedback results in an auto-amplification positive feedback of Rac and Cdc42 (via 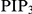) on themselves. At an intermediate level of PI feedback, this leads to lower, rounded peaks with higher shoulders. For example, 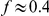 corresponds to smoother, more realistic profiles for the active forms of small GTPases (Fig. 3c, right). It also leads to growth in the heights of the active Cdc42/Rac peaks at the expense of the inactive forms. This creates a local depletion of the inactive GTPases, and substantial gradients in their availability i.e. growing activity peaks can ‘rob’ one another by depleting this pool (Fig. 3b, right). In presence of excessively high PI feedback, this can lead to highly localized peaks of GTPases with flattened tails. The stronger the PI feedback, the stronger the kurtosis of GTPase peaks. This results in concurrent depletion of the inactive GTPases as more and more activity is turned on locally at the expense of the global pool of available inactive forms.

Peaks of the active small GTPases Cdc42 and Rac correspond to zones of activity that spawn nascent lamellipodia. Hence, the communication of such peaks has an important influence on competition of protrusions. Complex stimuli can lead to multiple zones of protrusion. Without PI feedback, since active zones are flat plateaus that hardly interact, multiple peaks merge on an exponentially slow timescale [35], too slow for biologically relevant resolution of competing lamellipodia. This will be discussed further in the section on V-shaped gradient stimuli below.

As shown in the right panel of Fig. 3c), with appropriate feedback from PIs, zones of Rac activity communicate spatially through their augmented depletion of inactive Rac (similarly for Cdc42). Large-scale gradients of inactive cytosolic GTPases are formed as a result of the intense local exhaustion. This means that the spatial scale of communication is governed by the relatively fast effective cytosolic diffusion of inactive GTPases, rather than by the significantly slower diffusion of the membrane-associated active forms. This implies that communication between competing peaks of active GTPases is accelerated by 100–500 fold due to feedback from PIs.

We find that the modulating influence of PIs depends on the right balance between enough feedback for auto-amplification to enhance peaks of GTPase activity, versus excessive feedback that causes overly dramatic kurtosis of those peaks. If the magnitude of the feedback is tuned to values closer to 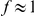, the resulting Rac and Cdc42 peaks become sharper and more highly localized, with resultant aberrations in cell behaviour (described below). Similar tuning of other parameters has the same consequences. Increasing the kinetic parameters of PIs (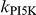, 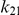, 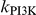, 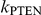) or decreasing the rate of diffusion 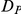 has similar outcomes (results not shown). The availability of inactive small GTPases and factors that influence this similarly play a role. Such factors include availability of GDIs, and their efficacy at extracting inactive small GTPases from the plasma membrane, which affects an effective rate of diffusion of these proteins [34], [36]. The longer the inactive GTPase spends on the membrane, the smaller this rate of diffusion, and the more significant are the effects of depletion described above. Note that, in contrast, without PI feedback the effective rate of diffusion of the inactive forms only plays a very marginal role, as long as that rate is at least a few times higher than the diffusion rate of the active form. This is due to the fact that without PI feedback the GTPase levels at the flat plateaus are not limited by the diffusion of the inactive form.

There is a fine balance, however, between sufficient and excessive autoamplification due to PI feedback. When 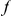 is too low, as already discussed, the competition of zones of high GTPase activity takes too long to resolve. Having 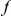 values closer to 1 leads to rapid resolution of competing peaks of GTPase activity, but at the same time, this also tends to “freeze” single peaks, reducing the ability of cells to respond by moving or turning. Consequently, we have found that the most effective strategy for cell motility is to adopt some intermediate level of feedback 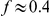.

### Effect of cell shape and geometry on dynamics

There are two distinct geometric factors that affect dynamics: the shape of the cell and the geometry of isoclines. Since actin remodelling is a direct consequence of the signalling system, it is clear that the shape of the cell is downstream of the signalling modules, so this direction of influence is obvious. The possibility that there is feedback in the opposite direction, from cell shape to signalling biochemistry is more subtle. Here we show that cell shape also influences the biochemical kinetics through a geometric (rather than hard-wired) effect. This implies a feedback loop between cell shape and intracellular dynamics with important consequences for cell behaviour, such as rate of turning towards an external signal.

Mathematical investigations of reaction-diffusion systems have shown that geometry and dynamics are linked. In many systems, it is well known that curvature of a moving interface can locally accelerate or retard its motion [42]–[44]. The shape of the boundary of a domain, and the conditions imposed at those boundaries (e.g. impermeability), put constraints on the possible behaviour. For example, no-flux (also called Neumann) boundary conditions (BCs) imply the orthogonality of chemical isoclines at points of intersection with the boundary. Given that the cell boundary is nonpermeable for lipids and proteins forming the signalling system, and given that the keratocytes that have been used as a paradigm system for this study have a very flat, almost two-dimensional shape, no flux BCs hold in our model for the GTPases, PIs and signalling components and their isoclines are therefore always perpendicular to the cell edge. As this constraint holds at any time 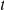, it implies that isoclines bend or rotate whenever the boundary deforms locally, preserving that orthogonality. In the case of a dynamic cell shape, e.g. when the cell turns or reorients, the regions of locally high curvature on points of the boundary result in deformed and highly curved segments of the isoclines, including the front-back interface discussed above (that interface is itself an isocline.) As a result, the effects of heightened curvature drive accelerated dynamics and result in a faster biochemical response.

### Cell shape and interface minimization

**Figure 4:**
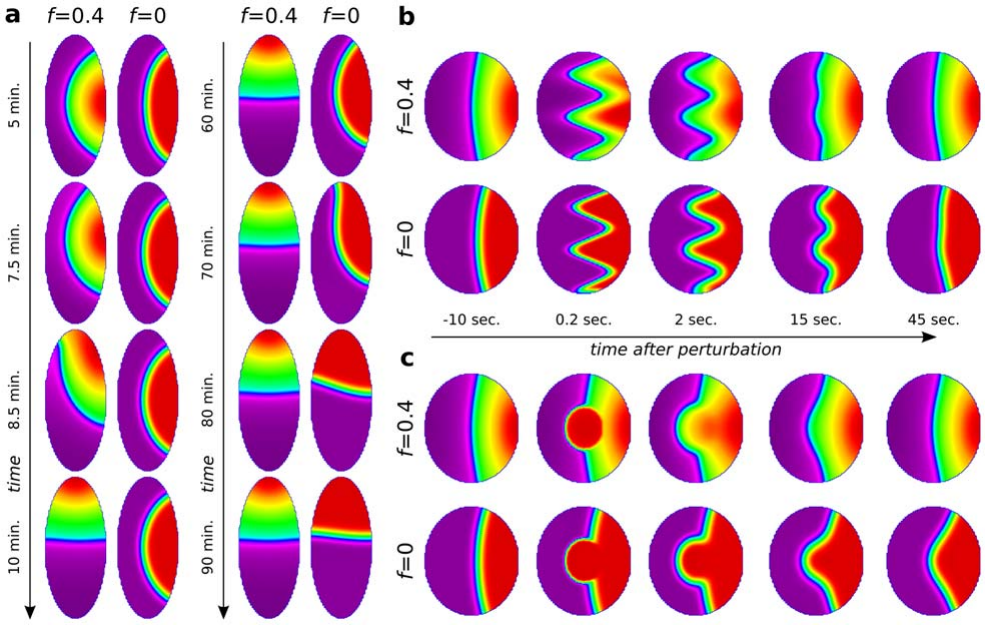
Effect of cell shape and intracellular ‘front-back’ interface curvatures. Shown are Cdc42 distribution profiles at indicated times using the colour scheme as in Fig. 2. (**a**) (and Video S2) Effect of cell shape on repolarization in the presence (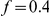, left) and absence (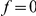, right) of feedback from PIs to Rho GTPases. Cells with elliptical, static shape are initially polarized along their short axis using the standard, transient gradient protocol. Due to their shape alone (with no further stimulus or bias), there is a clear tendency for the cells to repolarize. The dynamics of shape-induced repolarization occurs much more rapidly when PI feedback is included. (**b**) Static circular cell in which the intracellular profiles have been modified into a highly curved profile. Over time, the curvature of the front-back interface flattens (with regions of higher curvature changing faster). (**c**) Local injection of Cdc42 appears to locally distort the intracellular interface, which then straightens again. Shape-sensitivity and robustness to local perturbations can be understood through the tendency of the reaction-diffusion system to minimize the front-back interface. The no-flux boundary conditions further assure that the all level curves (interfaces) maintain right-angles to the cell membrane. For the dynamics of (b,c), see also Video S3.

First we analyze the feedback between cell shape and intracellular polarity by uncoupling the dynamics of the cell shape changes from the dynamics of the internal biochemistry. We did this by studying the effect of the shape of the cell perimeter in immobilized cells with PI feedback (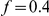) and without (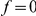), as shown in Fig. 4a and Video S2. (Immobilized disk-shaped cells can be obtained *in vitro* using latrunculin, e.g., see [45] and others.) Specifically, we asked how confining the cell to a specific, immobilized elongated shape would impact the chemical polarity in each case. We use an immobile ellipsoidal cell, initially polarized along its shortest axis by means of an external signal. Note that if the cell is polarized along its shortest axis, the front-back interface is parallel to the longest axis. Once the applied stimulus gradient is turned off, the chemical distribution (but not the cell perimeter) is allowed to evolve. Interestingly, the direction of polarity spontaneously reorientates to align itself along the longest axis of the cell, thereby minimizing the length of the front-back interface, which becomes positioned along the shortest axis. With PI feedback (Fig. 4(a), left), the broad region of “frontness” in the cell rapidly relocates to the pole of the ellipsoid. Without PI feedback (Fig. 4(a), right), the interface also decreases, but spatial coupling is much weaker. Hence, a globally optimal configuration of the active zone is only attained after an excessively long (biologically unreasonable) time scale. Note the difference in timescale for repolarization with PI feedback (within 10 min) and without it (more than 90 min) (see also Video S2). Again there is an optimal feedback strength, because when 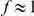, the amplification caused by the PIs becomes too high, causing a freezing of the initial polarization and a complete failure to reorient (results not shown).

To dissect this process of interface minimization further, we purposely initiated a circular cell with an irregular “wavy” front-back interface. Fig. 4b illustrates how the curved interface straightens, with highest curvature regions changing most rapidly, so that the overall length and curvature of the interface decreases (see also Video S3). Again, this process is significantly faster when PI feedback is included (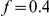, top row), than when it is absent (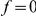, bottom row). Similarly, *in silico* experiments of “micro-injecting” active Cdc42 in the middle of a polarized cell have a similar effect (Fig. 4c; Video S3) (see Materials and Methods for details). This results in a perturbed interface, which then rapidly reestablishes its flattened geometry. These results together illustrate the PI-enhanced tendency to shorten and flatten the border between the front and the back of the cell.

The observation that biochemical kinetics coupled to diffusion can drive the length minimization of the interface between two stable states within a system is known to mathematicians. This fact can be explained by the following argument, as pointed out by a reviewer of this paper. Reaction-diffusion systems with multiple steady states can be represented as gradient flow problems with an assigned effective energy. This “energy” depends on the reaction terms as well as on a gradient squared term that captures the diffusion terms. In such a representation, the stable states of the dynamical system are given by the minima of the reaction “energy”. Given that the gradient flow acts to minimize the energy, the configuration of a spatial system with a fraction in one stable state and the rest in another stable state will therefore evolve so as to continuously reduce the length of the transition region (i.e. to minimize the integral of the gradient squared of the concentration). (See, e.g. [46].) Note that in this study an extra complexity arises because the two “steady states” are a consequence of the fast diffusion of the inactive forms, i.e. the reaction part does not itself entail multiple steady states. Nevertheless, the argumentation underlying the interface length minimization still holds.

### Cell shape feeds back on interface dynamics

**Figure 5 pcbi-1002402-g005:**
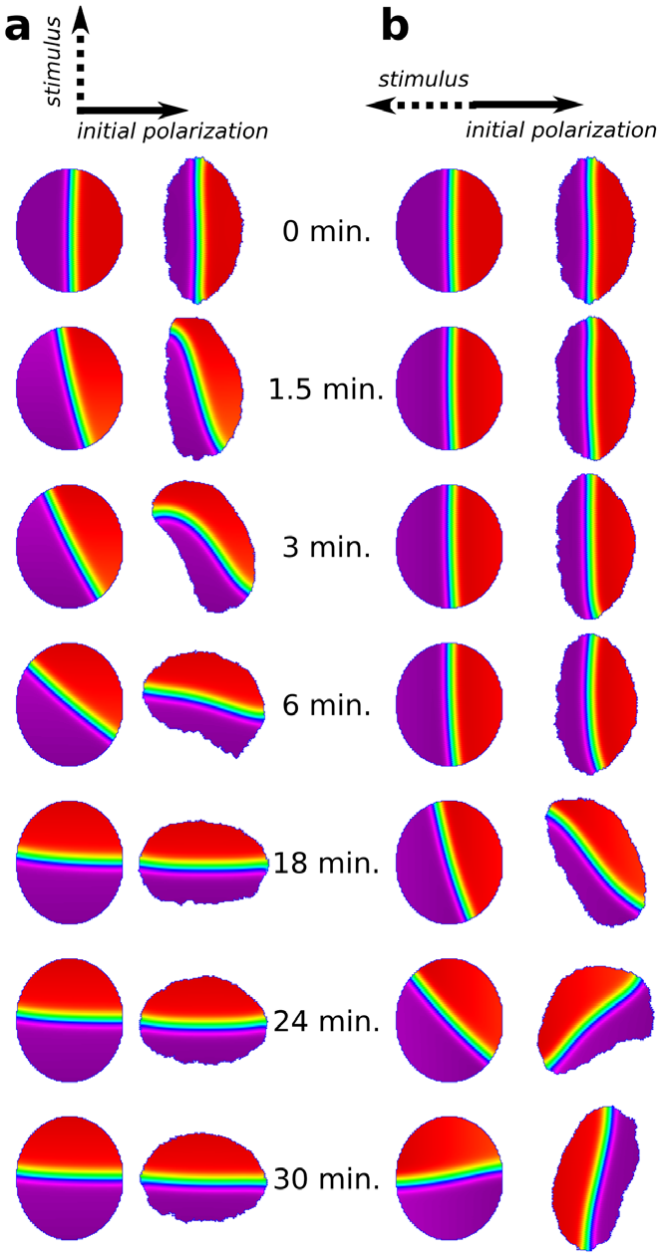
Feedback of cell shape dynamics on intracellular dynamics. (**a–b**) A comparison of Cdc42 repolarization in a cell whose shape is frozen (left columns in each sequence) with a control cell that has a dynamic shape (right columns in each sequence). (See also Video S4.) In both cases, the cell is initially polarized by a transient gradient, then repolarized by either an orthogonal (a) or opposing (b) second gradient. Images on the left and right were taken at the same times after the second stimulus. Note that in (a), after 3 min there is a noticeable difference in the cell's polarity as seen from the angle of the front-back interface with the stimulus gradient; the difference is accentuated even further by 6 min. In (b), the static cell only partially repolarizes during the time span when the control cell has completely repolarized. The evolving cell shape spontaneously twists the intracellular interface which has to maintain its orthogonality to the cell edge. The increased curvature of this interface has a faster rate of flattening, driving the chemical dynamics to adjust more rapidly. As the chemistry also feeds back to protrusion/contraction and shape change, the two-way feedback resulting from cell-shape dynamics leads to a faster overall turning and aligning with the repolarization cue.

As noted above, cell shape influences the dynamics of signalling even when the shape of the cell is static. But signalling cascades also cause the shape of a cell to evolve (unless specifically blocked as in the previous test). Thus, cell shape and signalling concurrently influence one another. Here we aim to illustrate the effect of this feedback. Fig. 5 demonstrates the effect of cell shape changes on the internal dynamics. We here contrast the speed with which repolarization occurs in an immobilized cell with static shape (left sequences in Fig. 5a,b) versus a cell in which shape is dynamic (right sequences in Fig. 5a,b). Cells were first polarized using standard protocol with a gradient of 10 s duration. At 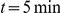, a new gradient of smaller magnitude was introduced at 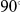 (Fig. 5a) and at 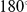 (Fig. 5b) to the original gradient. For both angles, motile and static cells detected and chemically repolarized to the new gradient, i.e. were capable of changing their directionality to track the new cue. (Even for the extreme angle of 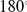, motile cells performed a “U turn” to align to the new gradient, as described in the literature, e.g. by [47].) However, the speed of repolarization is significantly faster in a cell with dynamic shape. The most noticeable acceleration of repolarization is obtained in cases where cell shape changes induce the most dramatic curvatures in the front-back interface (see also Video S4). For example, during the U-turn, with shape-dependent feedback the cell is able to turn 90 degrees within 10 minutes (Video S4, from 13:00 till 23:00), while without feedback during the same period the rotation is only 30 degrees.

The feedback from membrane curvature to local biochemistry, and hence to overall polarity, is quantitatively dependent on the magnitude of the curvature changes in the cell shape during the turning of the cell. The strength of the feedback will therefore be more prevalent under conditions that favor more dramatic cell shape changes, basically when the cell interfacial tension is low. Thus, when the membrane coupling energy (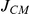) and/or membrane stiffness (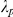) are lowered, the cell deformations become more extreme, and hence the feedback becomes more pronounced (see Video S5).

The changing cell shape results from actin dynamics described previously. Importantly, no additional feedback from actin to PIs or small GTPases has here been assumed explicitly in the model. Rather, the dynamic shape itself leads to a faster chemical repolarization. With the above observations, we are now in position to understand the results of further *in silico* experiments.

### Resolving complex stimuli

As shown above, when there is a single front and back, the interface separating these is linked to the cell shape and to the dynamics of the leading edge. Here we asked what happens when there are multiple interfaces, due, for example, to many “fronts” that form spontaneously. To address this question, we challenged the model cell with a variety of stimuli to investigate its ability to resolve multiple conflicting cues, comparable to *in vitro* experiments. Details of the stimulus protocol are given in the Materials and Methods.

#### V-shaped gradient stimulus

**Figure 6:**
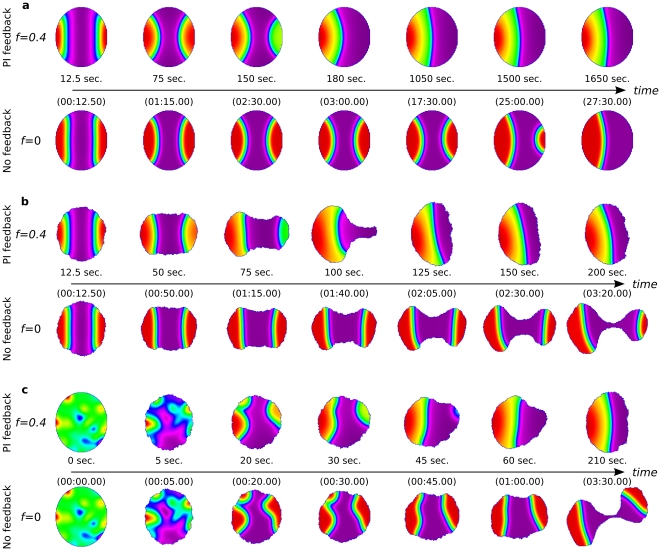
Resolving conflicts. Effect of phosphoinositide feedback (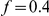) versus absent feedback (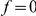) and cell shape dynamics on the response of cells to competing, conflicting, or noisy stimuli. (a) “V” shaped gradient with static cell shape. (See Video S6). (b) “V” shaped gradient with dynamic shape changes. (See Video S7). (c) Noise-induced fronts within motile cells. (See Video S8). PIs allow the cell to rapidly resolve the competition between contradictory signals.

Previously [36] we showed that cells can respond chemotactically to even very shallow external gradients. For example, a gradient over the cell diameter of 0.5% in terms of the Cdc42 basal activation rate is immediately sensed by the cell, causing rapid and complete turning towards the gradient. Even gradients of one tenth this magnitude can elicit turning responses. Here we ask how the cell copes with multiple stimuli. We stimulated the cell with a symmetric, transient V-shaped gradient to promote conflicting directional cues (see Materials and Methods). In Fig. 6a and Video S6, we compare the response of immobilized cells with (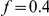) and without (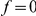) feedback from the PIs. In Fig. 6b and Video S7, we do the same for cells with dynamic shapes. In all cases, the initial stimulus creates two regions of “frontness” in the cell. When the stimulus is removed, these zones vie for dominance.

Curvature of the isoclines is an important influence in that competition for dominance. As demonstrated in the previous results, regions of activity with more curved isoclines shrink more rapidly than those with flatter isoclines. But larger “frontness zones” maintain interfaces with lower curvatures (see, e.g. the cell in row 2 at 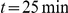). Thus, these zones tend to grow at the expense of the more highly curved, smaller “fronts”. As shown in the top rows of Figs. 6a,b, with PI feedback, one zone rapidly takes over at the expense of the other, minimizing the global interface length and curvature. The autoamplification caused by PI feedback and depletion of inactive GTPases combines with the effect of curvature of the interfaces. This means that larger peaks of active GTPases are able to more rapidly deplete the global pool of inactive GTPases, at the expense of the smaller peaks of activity. The result is a “winner takes all” phenomenon, wherein redistribution, and selection of a unique front, is accelerated up to 10 fold due to the PI feedback. It means that within a short, biologically reasonable time, the conflict is resolved. As before, we note that immobilized cells (Fig. 6a) take longer (up to 180 s) to select a single direction, whereas cells with dynamic shapes do so faster (by about 100 s).

Where feedback from PIs is missing (rows 2 and 4), the curvature of the interface is the dominant influence. In such cases, the competition continues for much longer. Cells with dynamic shape (row 4) are undecided by 200 s, tending to stretch into dumbbell shapes and break apart. Immobilized cells take up to 1650 s to resolve conflicting fronts (row 2).

We can understand the results of these experiments as follows: In a perfectly symmetric V-shaped gradient, slight stochasticity makes one putative lamellipod slightly larger than the other by chance at some instant. This spawns slightly more actin branching, local buildup of F actin in that lamellipod, and thus positive feedback on further broadening on that side. Slight growth thus reinforces growth of one of the two lamellipods by autoamplification.

A larger lamellipod tends to protrude and extend outwards more easily. This leads to a translocation of its “leading edge” outwards. A similar effect takes place at both nascent lamellipodia, stretching the cell into a “dumbbell shape”. For each lamellipod, the front-back interface would tend to track its protruding edge, but when two lamellipods compete, the interfaces cannot maintain the same speed as their respective leading edges, since they share a common pool of inactive GTPase. The retraction of the interfaces then becomes equal and opposite, each moving with some intermediate speed. The expansion of one lamellipod is at the expense of the other: the smaller lamellipod continually loses a fraction of its active GTPase to the larger. We tested the cell with several other protocols, including microinjection of active Cdc42 at two poles of the cell. Results were essentially analogous in such cases and are omitted due to space constraints. With feedback from the PIs, the stronger global coupling of curvature and area is evident. The lamellipod with larger area tends to dominate to an even greater extent. In that case, the effect of interface curvature, though present, is more subtle to observe.

In simulations above, we challenged the cell with graded stimuli of opposite directions (i.e. at 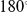 relative angle). In general, the outcome depends on the angle bewteen the gradients (results not shown). When that angle is smaller than 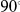, the cell moves towards the integrated mean of the two applied gradients. The precision of the motion is closely linked to the timescale on which the motion is observed: on a short timescale, the cell takes on a ‘wiggly’ motion, due to underlying stochasticity of the CPM. At a longer timescale, the direction of motion becomes very precisely determined by the mean gradient, and independent of the underlying computational grid. However, when the angle between the gradient directions is larger than 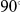, the cell can no longer integrate the information into a combined, averaged outcome. Rather, the single direction that is chosen depends on the strength of the competing gradients. When the gradients are very comparable in magnitudes, the initial stochasticity eventually plays a large role in the direction selected. Thus, PIs play an important role in preserving the integrity of cell polarity despite opposing stimuli that would otherwise spawn multiple leading edges and break up the cell.

#### Noise and spontaneous polarization

When activation is induced in a noisy fashion, it may happen that multiple regions of ‘front-activity’ (i.e. high Rac, Cdc42) arise within a cell. We explored the effect of inducing polarization in this manner for motile cells with feedback (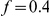) and without feedback (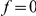) from PIs (Fig. 6c and Video S8). We found that the time-scales on which the emerging noise-induced fronts resolve to form a single leading edge is critical to maintain the integrity of the cell. In the presence of PI feedback, the multiple patches of high Cdc42 concentration quickly ‘merge’ (within a minute), so that the cell motion is quickly coordinated in a single direction. In the absence of PIs, it can happen that fronts form in extreme regions of the cell, and given that the dynamics of their fusion is slow, downstream effects emanating from the front-regions to the cytoskeleton result in multiple extensions, which lead to drastic cell deformations. This deformation of the cell shape only increases the distance between the front-regions, exacerbating the difficulty of resolving the conflict between the emerging fronts. Thus, these *in silico* observations highlight the importance of quick global polarity coordination in relation to the dynamics of the downstream effects on cell shape.

#### Interaction of cells with obstacles

It is also known that for single motile cells, as well as for cells within multicellular developmental contexts, mechanical signal transduction pathways downstream of integrin receptors allow for a variety of responses to mechanical stimuli. Here such pathways are not included, so all behaviour reported below is independent of direct mechanical signalling. However, we wanted to explore the role of geometry and feedback between shape and biochemistry and what effects result solely from these interactions when cells encounter mechanical barriers. We thus proceeded to challenge cells with obstacles and asked whether the regulatory network would enable the cell to navigate around a barrier, or to reorient to crawl along a wall that it encounters.

**Figure 7:**
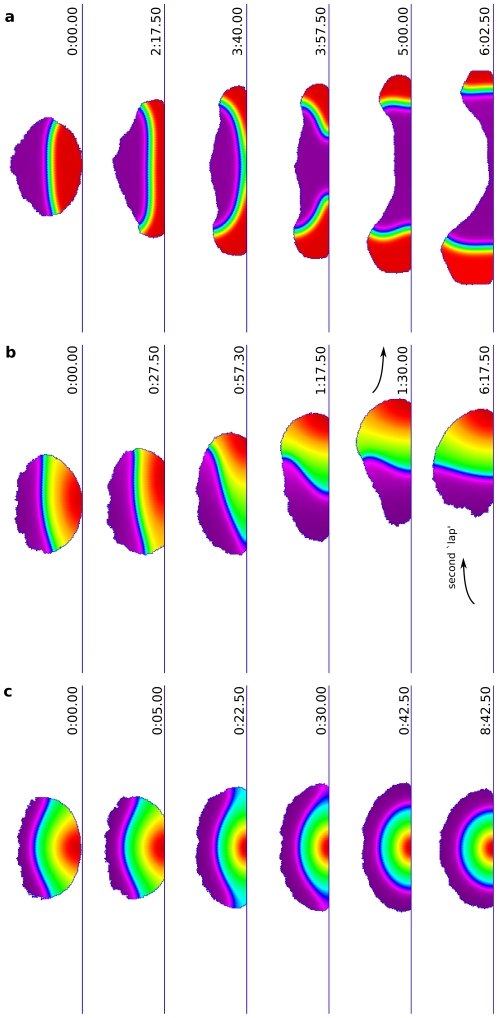
Response of cells with varying degree of PI feedback to a wall. Cells initially polarized and moving rightwards encounter a wall (blue vertical band). (**a**) without feedback from PIs (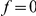) the cell has difficulty resolving the competition between two nascent “fronts”; (**b**) with the standard intermediate feedback from PIs (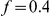) the cell is able to make a decision and move smoothly along the wall; (**c**) with extreme feedback from PIs (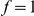) the leading edge becomes confined and encircled by the contracting back, so that the cell becomes stuck. (See also Video S9.)

In Fig. 7a we show a polarized motile cell with no PI feedback approaching a wall. The leading edge of the cell flattens and extends parallel to the wall. Concomitantly, the zone of high Cdc42 (and Rac) activity shifts from the front edge to the ends of the cell, by the same interface-shortening and curvature reducing mechanism previously noted (see also Video S9). Growth and protrusion at these two unimpeded ends of the cell cause a retraction of the chemical interface, creating two independent peaks, one at each pole. The situation is then comparable to previous examples of the tug-of-war between two active Cdc42 (Rac) maxima. In many cases, this leads to the breakup of a cell.

When PI feedback is absent, the double-lamellipodia cell that forms at the wall occasionally resolves (for angles of incidence other than 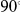), into a polar unidirectional cell. Even with a barrier at 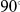 to the cell's direction of motion, in some cases a successful decision is made to navigate up or down the wall, but only after a significant delay (simulations not shown). These observations point to great difficulty in overcoming the challenge of a barrier when PI feedback is absent. With PI feedback (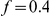), Fig. 7b, the resolution is easier, and almost no conflict is apparent. The cell rapidly and easily translocates its leading edge to one or the other direction and glides smoothly along the wall.

When the PI feedback is too strong (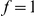), causing overly high amplification of the Cdc42 (Rac) peaks, those peaks become highly focused, and fail to relocate (Fig. 7c). Inactive GTPase then becomes so depleted that other places are unable to build up activity. We then see stagnant cells that have become confined and immobilized at the wall, overwhelmed by their own Rho-induced myosin contractility. This same effect also interferes with a cell's ability to reorient to a new gradient, illustrating again the balance needed in the level of PI feedback.

**Figure 8:**
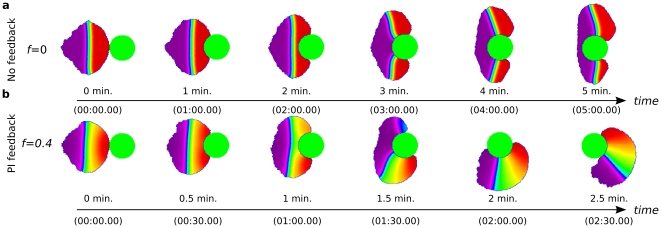
Response of a cell with and without PIs to obstacles in its path. As in Fig. 7, the cell is initially polarized and moving to the right until it encounters an obstacle (static green circular object). (**a**) Without feedback from PIs (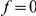) the cell has difficulty resolving the competition between two nascent “fronts” which embrace the object. (**b**) The cell with feedback from PIs (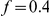). The cell is able to move around the object smoothly. (See also Video S10.)

When cells are challenged by smaller objects directly in their path, we also observed similar behaviour (Fig. 8, Video S10). When PI feedback is absent (and with an identical initial configuration), the response to the obstacle becomes conflicted. Two competing lamellipods are formed, and the cell is stretched and pulled in opposing directions, unable to reach an appropriate decision (Fig. 8a). In contrast, with PI feedback, the cell smoothly selects a path and navigates around the obstacle. A unique and stable lamellipod guides this motion, with hardly any evidence of conflict. In some cases, several circuits are made around the obstacle, until eventually, due to a small stochastic adjustment of direction, the cell moves off along a tangent, and breaks free.

## Discussion

The famous *in vitro* clip of a neutrophil navigating between red blood cells while chasing a bacterium reinforces our intuition that real cells encounter complex environments where multiple decisions and rapid changes in orientation are essential. This is even more dramatic *in vivo*, as shown, e.g. in [8]. To understand how cells can respond to such cues, we here simulated actin-based cell motility and its regulation by small GTPases, modulated by feedback from the phosphoinositides. The main motivations were (1) to explore the role of PIs in fine-tuning direction sensing in response to complex stimuli and (2) to demonstrate the bi-directional feedback between the signalling modules and the dynamically evolving shape of the cell.

We explored a **biophysical feedback**, wherein geometric effects, and cell shape affected the biochemical dynamics. When local protrusion occurs, the cell perimeter becomes extended and curved, causing chemical isoclines to be curved. The curvature-reducing effect of the reaction-diffusion dynamics then speeds up the response significantly relative to the basal rate of polarization for a cell with static shape. The internal chemical pattern, in turn, specifies the sites where actin nucleation and growth will lead to protrusion of the cell edge, and affect the isoclines yet again, closing the feedback loop between biochemistry and cell shape. While the importance of morphology and cell shape has been discussed in other papers [48]–[50], here we have incorporated full dynamics changes in both shape and chemical distribution, allowing for feedback in both directions.

The implication of this finding is that cell shape is *not just a downstream consequence of regulatory pathways that impinge on the cytoskeleton but rather, an integral part of the feedback mechanism*. Papers in the literature have suggested that actin filaments feed back onto PI localization. Here we have shown that part of that feedback could stem directly from the changing cell shape, and not only from a direct interaction between actin and PIs. Mechanical effects (e.g. via integrin signalling not here considered) would substantially magnify such purely geometric effects. As shown in Figs. 4–6, a frozen cell shape with absence of feedback from PIs to GTPases takes up to 10 times longer to respond to a repolarizing signal, or to decide between conflicting cues. Cells with dynamic shapes respond more quickly, and those with PI feedback in the appropriate range 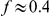 (not too high, and not too low) are even faster.

A second theme in our paper concerns **chemical feedbacks**. In our simulations, decreasing the parameter 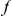 (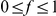) corresponds to a gradual PI3K silencing (e.g. as in [8] with the PI3K inhibitor, LY294002) and we have here examined the effects of tuning this parameter between overexpressed PI3K to full knockout. We have shown that both extremes are pathological, so that wild-type behaviour resides at some optimal level of feedback (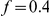). In particular, peaks of Cdc42/Rac activity tend to be platykurtic when PIs have no feedback (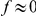), leptokurtic at high levels of feedback (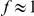). In the former case, communication between plateaus of activity is restricted. In the latter case, shoulders are broader, and so the zones of activity interact directly. Autoamplification due to PI feedback also raises the magnitudes of the activity zones, and depletes the inactive GTPases, leading to longer-range global communication and depression of competing peaks.

We found that PI feedback works optimally at some intermediate level. At that level, it can help to speed up the response to new stimuli and to resolve confusing or contradictory external cues. As PI influence is tuned to higher intensity (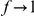), the ability to displace a peak of active Cdc42 (Rac) decreases, but the ability to resolve conflicts by a “winner take all” mechanism increases. A too-high PI feedback is inappropriate for motile cells exposed to challenges such as conflicting stimuli or obstacles. We showed that if PI feedback is too strong, cells get pinned to an obstacle or face difficulty in reorienting to new cues. (This would present serious challenges in the complex environment of a living tissue.)

The optimal level of feedback from PIs depends on the type of cell and its function, and whether multiple peaks of Cdc42 or a single peak is needed for some cell function. Plant cells have ROPs, which are analogues to the small GTPases of the Rho family [51]–[56]. For example, ROP 2 and 4 play a role similar to Cdc42, defining a “leading edge” zone whereas ROP 6 in plants is analogous to Rho in animal cells. In pavement cells of plants, for instance, multiple lobes are a functionally important feature to allow cells to interlock like jigsaw puzzle pieces. Hence, multiple static peaks of small GTPase activity are observed, suggesting that a different PI feedback would be optimal there: either higher or much lower. For 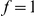, we observe firm immovable peaks of GTPase activity, and for 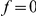 we found that distant peaks hardly interact. Such extremes would possibly fit the plant repertoire more closely. Also, the specific details of the small G-protein crosstalk can significantly differ between different experimental systems, and this can influence both biophysical and chemical feedbacks. For example, using biosensors for the three Rho GTPases and mouse embyonic fibroblasts (MEFs), Machacek. et al. [57] showed that Cdc42 activates Rac1, as is assumed in this study. They, however, find mutual inhibition between Rac1 and RhoA, and, contrary to older work by Bourne's lab, they find that RhoA gets activated right at the advancing cell edge, and that Cdc42 and Rac1 are activated 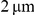 farther back with a delay (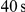). It remains to be seen to what extent such findings are cell-specific or wide-spread.

By exploring this model, we gain several **insights** that help to understand the biochemistry. For example, the importance of GDIs emerges from our analysis of factors that influence communication of activity peaks. We argued that one such factor in peak communication is the “effective cytosolic diffusion” of inactive small GTPases. Upregulating GDIs extracts inactive small GTPases from the cell membrane, effectively increasing their diffusivity. Downregulating GDIs means that inactive small GTPases spend more time on the membrane, and have smaller diffusivity [58]. Similarly, increasing the kinetics of the PIs (equivalent to up/down regulating PTEN, PI3K, etc) produces an analogous tuning of the interpeak communication. Such parameters are tuneable outcomes of evolution, with species-specific and cell-type-specific variability. A range of dynamical effects would thus be expected in control and mutant cells, or cells treated with inhibitors or drugs.

Recent reviews of the models for eukaryotic chemotaxis and their relation to experiments include [33], [59]–[61]. Existing models based on Rho GTPase and/or PI signalling [48], [62]–[67] are mainly concerned with explaining polarization. Other theoretical models [68]–[71] describe circuits with capability for adaptation, direction sensing, or polarization. Previous models for 2D cell motility include steady state cell shapes [72]–[74] and evolving shapes using force-based methods [75], [76]. Recent computational models for cell motility have also been based on level-set approaches [77]–[79] phase-field methods [80], and other approaches [81]. Our model, based on energy minimization [40], [41] allows for rapid and convenient reaction diffusion of chemicals on an irregular domain, and for effective forces of protrusion and contraction that can be put into correspondence with real forces due to actin filament barbed ends and actomyosin contraction [36]. This method has the advantage of providing a good description of thermal-noise induced stochastic shape change of the cell edges, while affording speed and efficiency of computation. Such energy-minimization techniques have become more widely adopted for describing cells and tissues [82]–[84] because they can dramatically speed computation. The efficiency of the implementation allowed us to focus on exploring the response of the model cell to specific stimuli, with a variety of geometries. Future work should address a comparison of similar ideas in other 2D simulation platforms. We anticipate that results discussed here would carry over universally to a variety of approaches for capturing the evolving shape of the cell.

Using a mathematical model, Meyers. et al. [85] considered the effect of cell spreading (and flattening) on rates of (de)phosphorylation due to proximity of the plasma membrane to cytosolic intermediates. They noted that this effective change in activation/deactivation rates links cell size and shape to regulation of signaling pathways. While they were concerned with the “thickness” dimension of motile cells (that we take to be constant), we are here describing the effects of curvature and 2D cell boundary shape on the dynamics of interfaces of the internal RD system.

Model limitations include absence of direct mechanical forces and integrin signalling. Thus, this model would not be appropriate for describing keratocytes “bouncing” off walls they encounter, or cells following mechanical cues. The pathways and rate constants used for the signalling module could be variations on specific versions at play in specific cell types and conditions, but behaviour was robust to modest changes in most parameter values. In [35], we showed that far simpler GTPase circuits (consisting of a single GTPase in its active and inactive forms) can already account for polarization reinforcing our belief that such overall dynamic motifs could operate in a more universal setting. Other cases where mutual inhibition between Rac and Rho are dominant would retain many of the overall features described here, while differing in subtle details, as do cells of distinct species. Also, the model does not capture possible effects of fluid convection in the cytosol (see Material and Methods for details on our implementation of moving boundary conditions). It would be interesting for future studies to address possible effects of intracellular convection by implementing reactant transport and developing a more complete description of the actin network, membrane and cytoplasmic flow of the moving cell.

While it has been shown experimentally that PI3K is not essential for chemotaxis in Dictyostelium discoideum, Yoo. et al. [8] found that PI3K is required for the interstitial migration of neutrophils in live zebrafish embryos. The mechanism of this effect was difficult to untangle. Our work in this paper highlights the fact the PI3K product 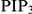 (and other PIs) facilitate the resolution of contradictory or multiple stimuli to the Rho GTPases. Such complex stimuli arise repeatedly as cells navigate through the complex environment of tissues (where blood vessels, other cells, or structures create obstacles that have to be circumnavigated).

The results suggest a number of important experimental investigations. First, although more data is becoming available, simultaneous measurement of the distributions of multiple GTPases and PIs in single cells is rare. Obtaining such correlated data would be valuable in characterizing the typical resting and stimulated states. Second, to check the effect of PI feedback, pharmacological inhibitors of PI3K such as LY294002 applied at successive level (very weak, to full inhibition), or mutants lacking PI3K could be compared with wild-type behaviour. To detect the differences, it would be important to challenge both treated/mutant cells and wild-type cells with multiple stimuli (as we have done *in silico*) or environments with obstacles to be resolved. Third, to check the predictions about cell shape, one can compare responses of cells treated with latrunculin (where the actin cytoskeleton is disrupted so that cell shape does not change) with untreated cells. When both are subjected to the appropriate time-varying stimuli, it would be possible to test our prediction that shape provides an additional feedback to speed of repolarization. As this effect can be subtle, one would need stimuli that lead to dramatic shape changes in the untreated cells to detect a substantial difference.

## Materials and Methods

In this section we summerize the model equations and parameter values, and we comment about our simplifying assumptions and implementation decisions. Details of the overall strategy for modelling and justification of the assumed crosstalk is provided in our previous papers [25], [34], [36]. In the latter two citations, models were in 1D, whereas here all variables are defined in 2D.

### Model equations

#### Small GTPase equations

The active forms of the small GTPases satisfy:
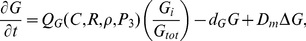
(1)where 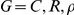 for the active forms of Cdc42, Rac, and Rho, respectively; 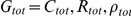 are the total concentrations of Cdc42, Rac and Rho, and 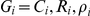 are the concentrations of the respective inactive forms. The inactive forms (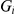) diffuse faster (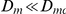) and satisfy:
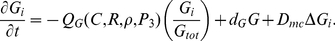
(2)The effective cytosolic diffusion, 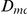 of inactive small GTPases is approximated by 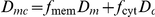 where 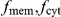 are the average fraction of time that an inactive molecule spends on the membrane and cytosol, respectively (influenced by the efficacy of GDIs).

The dynamics of the small GTPases on their own (when only their mutual feedbacks are being considered, i.e. only the top level of Fig. 1), are given by the following GEF-mediated activation rates for Cdc42, Rac and Rho:
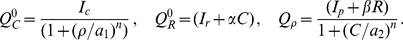
(3)


Here, 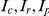 are the baseline activation rates, 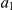 and 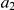 are the Rho and Cdc42 concentrations that elicit a half-maximal drop of Cdc42 and Rho activation, respectively. Here 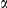 sets the rate of Cdc42 amplification of Rac and 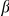 the rate of Rac-enhanced Rho activation.

To include the feedback from 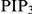 (intermediate level in Fig. 1) to activation of the small GTPases, we revise 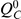 and 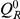 of Eqn. 3 to
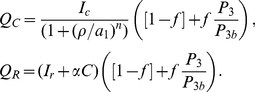
(4)where the parameter 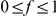 tunes the feedback from 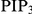 to activation of Cdc42 and Rac. Note that when 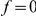, there is no feedback and terms in Eqn. 4 revert to those of Eqn. 3. (The activation rate of Rho is considered to remain PIP-independent.) 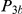 is the baseline concentration of 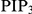 in a resting cell. The details of the functions are less important than their nonlinear sigmoidal shape, with 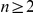. All parameters are as defined in Table 1.

#### Model of PI dynamics

The equations for PIs are as in [25] (but excluding direct feedback from actin to PIs):
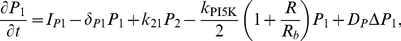
(5a)

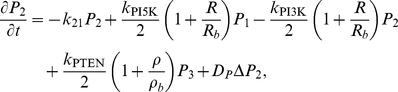
(5b)

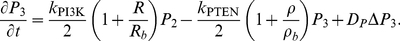
(5c)Where 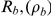 are typical levels of Rac (Rho) that result in a doubled kinase (phosphatase) activity level. Note that we implemented the simplest assumptions about feedback from GTPases to kinases and phosphatases and from PIs to small GTPases: i.e. these are assumed to be roughly linear processes, and the presence of saturation (which would introduce more parameters) was not needed. Ratios of the parameters 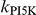, 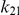, 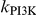, 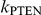 computed in [25] were based on steady state levels of PIs cited in the literature. Previous absolute values used in [25] are more appropriate for small (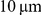 diameter) cells. These were amended to preserve appropriate spatio-temporal dynamics in a motile cell of size 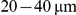 and 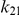 was changed to 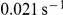.

#### Boundary conditions and their implementation

All diffusible substances (GTPases, PIs, Arp2/3) satisfy no-flux boundary conditions at the cell edge, i.e. for any concentration 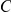 (representing one of these variables),
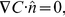
(6)where 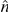 is a unit normal vector to the cell edge at the given boundary location. In our implementation, each simulation timestep consists of a reaction-diffusion step followed by one Monte-Carlo CPM timestep. During the CPM timestep we do not explicitly model the intracellular convection due to movement (which would depend on the cytosol's viscosity etc). This would however be a very important extension to such types of models, allowing possible effects of advection to be explored and analysed. Although we are currently ommiting convection within the model, we nevertheless ensure that (a) there is a conservation of molecules and (b) local extensions and retractions of the membrane are sufficiently small in comparison to the diffusion length of the intracellular chemicals to avoid large local concentration differences due to membrane movement. This prevents instabilities and amplifying feedbacks due to such possible simulation artifacts. Conditions a) and b) are met through the following implementation: when an intracellular pixel extends out into an extracellular site (i.e. the intracellular identity of a pixel is given to a neighbouring position that was previously part of the extracellular space), the new site receives the same values as the extending cell pixel (i.e. all concentration values are copied into the new position). This ensures a locally flat concentration profile. To then guarantee mass conservation, we renormalize all concentrations over the entire cell such that the total mass remains constant (i.e. dividing all concentrations by a factor corresponding to what has been gained or lost during one Monte Carlo timestep). We assure that timescales and spacescales are such that this correction is never larger than 0.1%, and hence is negligible compared to the diffusion processes.

We tested this implementation by comparing it with two alternatives. In one, we considered adding empty pixels when the cell extends and accumulating (‘heaping up’) concentrations when it locally retracts; this alternative approximates zero convection. We also tested a scheme where chemicals in an intracellular pixel are equally distributed between that pixel and its extended neighboring pixel. With the appropriate time step, we found that the dynamics of the cell behaviour do not strongly depend on the particular implementation. Thus we simply opted for the choice that confers the highest level of numerical robustness and therefore does not require prohibitively small timesteps.

#### Actin filament density and orientation

**Figure 9:**
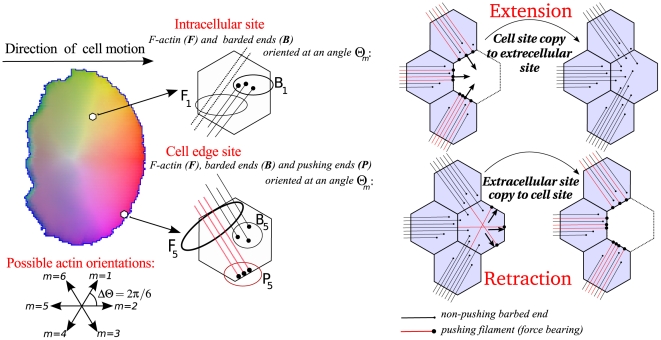
Membrane extensions and retractions. **(left)** Filament and barbed end densities are described at each grid point within the simulated cell (schematically shown by two representative hexagons), and for all possible orientations 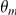, shown at the bottom left. Additionally, close to the membrane we specifically distinguish between barbed ends that have not (yet) reached the membrane (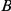) and barbed ends that are effectively pushing against the membrane (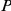), thereby contributing to the forces required for cell extension. **(right)** The CPM allows for cell shape changes and movement through updates corresponding to small site extensions and retractions. Here, these updates take into account the density of pushing barbed ends. When the cell extends, the pushing barbed ends increase the likelihood of extension (top right – note that all red filaments end up with a pushing barbed end and therefore contribute to the forward motion), while during retraction the barbed ends offer resistence, reducing the likelihood of retraction (bottom right – also note that many black (non force-bearing) filaments are promoted to red (force-bearing) filaments when the retraction is accepted).

Actin branching occurs at angles of 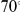 (due to the molecular configuration of Arp2/3-mediated sidebranching). We approximate this by 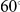 angles as it allows us to describe the distribution of actin orientations in the model by 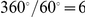 classes. This can be conveniently implemented on a hexagonal grid. Such a discretization leads to 18 coupled PDEs, given that we distinguish between filaments, barbed ends and pushing barbed ends (see Fig. 9).

With this in mind, the spatial simulations were run on a hexagonal grid, on which six filament orientations are modelled. For each discretized angles, 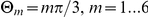, we model actin filament density 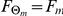 and barbed ends 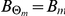 by
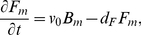
(7)

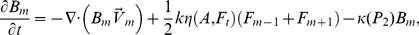
(8)where 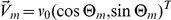 denotes the velocity of barbed end movement. Here 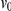 is the polymerization rate and 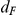 is the filament disassembly rate. Eqn. (7) describes the formation of F-actin by the polymerization at barbed ends (with rate 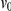) and filament turnover at a constant rate 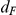. Eqn. (8) describes changes in barbed end density due to their flux throughout the domain, their nucleation at angle 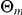 from parent filaments at angles 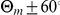 (via Arp2/3 branching), and their decay through capping.

Arp2/3 dependent branching of actin filaments (and nucleation of barbed ends) is assumed to be a saturating function of both Arp2/3 and F-actin. Capping of barbed ends are modelled as a basal rate that is reduced by 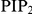:
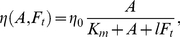
(9)

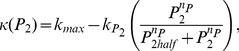
(10)where 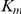 is a saturation constant. Note that the capping rate of barbed-ends is reduced by 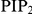 in a sigmoidal dependence with half-max parameter 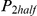 and Hill coefficient 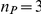. This means that at low 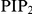 level, capping rate is approximately 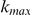, that it drops sharply past a threshold level, and that it is subsequently at the lower rate 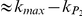.

Arp2/3 is modelled by
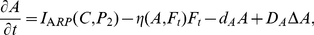
(11)where 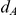 is inactivation, and 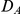 the diffusion coefficient. The activation rate of Arp2/3, 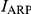, takes into account the (synergistic) effect(s) of 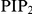 and Cdc42 as follows:
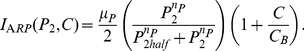
(12)


As barbed-ends move towards the membrane of the cell, they will influence the dynamics of the membrane. These force-bearing barbed ends, 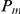, are those per unit edge length (in units of 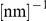) pushing the membrane at angle 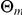,
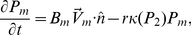
(13)where 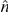 is the unit vector normal to the edge. Note that the variable 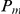 is defined only at the cell edge, and satisfies an ODE rather than a PDE. There is no divergence term in this equation, given that we are describing the net flux of filaments that would be locally crossing the cell edge if not prevented so by the membrane.

When the cell extends outwards by one pixel, of dimension 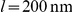, the distribution of pushing barbed ends that were previously at the edge are “demoted” to freely extending barbed ends, (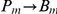). (See Fig. 9.) These now have to “grow” the additional distance to reach the new cell edge location. After some time, such new free barbed ends that are not capped will catch up with the protruding cell edge, since actin filament extension is much faster than cell movement. Then these barbed ends will be promoted back to pushing barbed ends, and will again contribute to forces exerted at that pixel. Similarly, retraction “promotes” barbed ends to pushing barbed ends (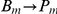). In both cases, we use a correction factor to capture the difference in units between barbed ends and pushing barbed ends.

### Stimulation protocol

The parameter regime we use here allows the cell to have both a stable rest state as well as a polarizable state. ***Standard Polarization:*** Polarity is initiated by applying a transient (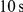) spatial gradient in 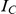 (the Cdc42 activation rate) with slope 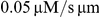, which corresponds to a roughly 15% variation across the cell. The gradient is then turned off. ***Repolarization:*** (Fig. 5) The cell is polarized as before. After 5 min, we applied a shallower second gradient, corresponding to roughly 3% variation in the Cdc42 basal activation rate (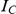 values) across the cell. The second stimulus was either orthogonal (Fig. 5a) or opposite (Fig. 5b) to the direction of the first. Shown are results for 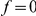. (Here turning is slightly faster than for 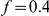, though qualitatively similar.) We used 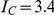 here (rather than 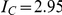) to slightly enlarge the front of the cell, and make the curved front-back interface visually more pronounced. ***Initial Predetermined Patterns:*** The intracellular distributions for the simulations of Fig. 4b were initialized by horizontally shifting a stabilized intracellular pattern in a sinusoidal fashion, such that all chemical species (PIs and (in)active forms of the small GTPases) have corresponding lower and higher levels, distributed in a sinusoidal pattern with three peaks. ***Injection:*** In the simulation of Fig. 4c, a spot of activation of Cdc42 was introduced by adding active Cdc42 concentrations at the highest level as found within the cell and diminishing this amount from the inactive pool. ***V-shaped Gradient:*** In Fig. 6a,b a V-shaped gradient across the horizontal axis (from right to left extremities of the cell) of 7.5% difference in 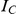 values was employed. (By a V-shaped gradient, we mean two superimposed, simultaneous, diametrically opposed gradients.) ***Noise:*** In Fig. 6c, the initial condition was a cell at steady state with uniform concentrations of all substances. On this we superimposed, only for the initial condition, normally distributed noise in the Cdc42 and Rac concentrations, with a spatial autocorrelation distance of 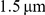 and a standard deviation of 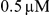, i.e. 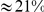 for Cdc42, and 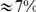 for Rac (see the first frame of Fig. 6c for a visual display of the initial noise level). A relatively high amount and/or spatially correlated noise is needed to push the cell out of the stable resting state. (We tested that continuously adding such a level of noise does not significantly change the results, as noise has only a small effect on the cell once polarity has been established.)

### The Hamiltonian and energy minimization scheme

Reactions and cell shape are computed on a 2D hexagonal grid. The cell, in a top-down view (approximated as having constant thickness) is represented as a set of pixel points on that lattice. Both cell interior (cytosol) and membrane are so represented, and chemical concentrations (in number of molecules per hexagonal cylinder of constant thickness) are tracked by implicitly solving the reaction-diffusion PDEs on the evolving domain. As the intracellular small G-protein and PI dynamics evolve, leading to down-stream effects upon the cytoskeleton, forces generated by the actin barbed-ends and myosin contraction change the cell's shape. To study the resulting cellular dynamics, and how these influence the internal chemical dynamics, we utilized a modelling framework in which membrane displacement is described according to an energy function. This is an approach that has recently become more widely recognized in modelling cell and tissue movement [82]–[84]. The core of this energy function includes biophysical properties such as cell adhesion, cell volume conservation, membrane and cortical tension, which together lead to an effective cell surface tension [83]. We utilize such an energy description within the Cellular Potts Model [40], [86] to describe the dynamics of the change of the cell's edge.

The Hamiltonian is defined by summing the energy contribution of each pixel over the entire field:
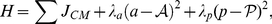
(14)(summed over neighbours up to 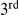 order). In 2D, 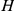 depends on cell area and boundary length (in 3D, on volume and surface area). 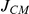 is the coupling energy per boundary site, 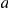 is the actual cell area, 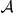 the target area, and 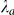 a parameter that describes resistance to deviation from the target area, 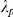 describes resistance to changing the perimeter 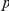 away from a target perimeter 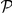. The perimeter constraint represents a high effective interfacial tension and energetic costs of stretching the cell membrane.

The dynamics of cellular movement result from the above Hamiltonian through the Monte Carlo simulation utilizing the Metropolis Algorithm, an energy-minimization method that allows the cell edge to change stochastically. Briefly, during each Monte Carlo step (MCS), each lattice site in the field will be evaluated in a random sequence. Sites at the cell's perimeter are queried for possible change to the state of a randomly chosen neighbour (“copying”). In the simulations here, in which we consider single cell dynamics, this local change implies protrusion or retraction of the edge of the cell.

The net change of energy due to a “copying” event, 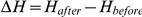 is computed, and the event accepted with probability
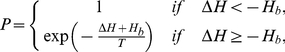
(15)where 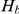 represents a yield, which is the ability of the membrane to resist a force, and 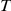 determines the fluctuations. Changes in state that decrease 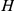 by at least 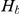 have probability 1, and other changes are made with a Boltzmann probability. Tuning the ‘temperature’ 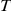 allows us to tune the magnitude of stochastic fluctuations (of various possible origins) in the model. For example, Mombach et al. [87] interpreted the parameter 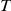 as the membrane fluctuation amplitude of cells, and they compared this with effects of the drug cytochalasin-B (a suppressor of membrane ruffling). Here, given that we describe the state of the cytoskeleton at the membrane, we are able to directly relate this parameter to the density and biophysical properties of the actin barbed ends.

Note however, that the cell is not expected to relax to a surface-driven equilibrium shape, as there are internal forces generated by the force-bearing barbed ends at the membrane. Thus, we describe these internal forces by altering probabilities of expansion/retraction dependent on the internal densities of barbed ends at the membrane as well as on the amount of myosin contraction as a downstream effect of Rho. Presence of barbed ends biases the probability towards protrusion, whereas presence of Rho GTPase biases towards retraction (see Fig. 9 for a schematic representation), and leads to the following forces at the membrane:
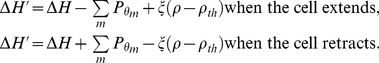
(16)

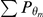
 describes the forces exerted by all barbed ends pushing against the membrane towards the empty site. The term 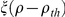 describes the effective Rho-dependent contraction forces when Rho exceeds the threshold level, (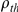). The term 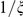 scales a unit of Rho elevation to the force of one pushing barbed end per nm membrane length. Note that according to Eqn. 16, 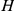 (and thereby 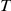 and 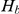) carry the same units as 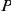, i.e. the number of extending filaments pushing against the membrane per unit edge length (here 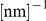). We can further relate the above expression to known physics of cell protrusion. An effective force-velocity relationship for protrusion speed as a function of the number of barbed ends pushing at the cell edge has previously been derived [37], [88]. In a thermal ratchet driven by actin polymerization, the relationship between the number of barbed ends at the membrane and the speed, 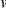, of the lamellipodial protrusion is approximately
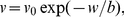
(17)where 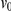 is the free polymerisation speed, 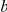 the density of barbed ends per unit length at the membrane, and 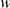 the renormalised membrane resistance force per unit length (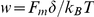, where 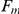 is the membrane resistance, 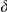 the size of one monomer, and 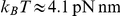 is the thermal energy). Neglecting capping and side-branching, and assuming that all barbed ends are directed normal to a straight cell edge, it can be shown [36], [41] that within the Cellular Potts Model Eqn. 16 implies a mean speed of protrusion
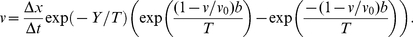
(18)Here 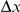 and 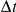 are the grid size and time step corresponding to one MCS, respectively. This is in line with Mogilner and Edelstein-Keshet [88]. While not identical to Eqn. (17), this equation also describes a relationship between protrusion velocity and the number of barbed ends. Here the relationship is expressed in terms of the CPM parameters 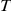 and 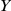. By fitting this relationship to Eqn. (17) (for which the parameter values are well-established), we obtained the optimal values 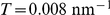; 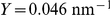. For these values, the thermal ratchet force-velocity relationship of Eqn. 17 and the effective force-velocity relationship of Eqn. 18 are highly comparable over the whole range of biologically relevant barbed end densities, which are typically observed to be in the range of 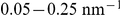 at the lamellipod edge [88], [89]. Accordingly, the CPM gracefully leads to a reasonable depiction of actin-based protrusion forces and the model quantitatively describes the response of the cell membrane to any possible load of pushing barbed ends. Having matched this relationship, we can now apply it in a simulation of a complex shaped 2D motile cell, with large variations in pushing barbed ends along the edge, implicitly locally solving for the large variation in the applied forces. This also allows us to determine the feedback between the cell shape and deformation on the underlying cytoskeleton dynamics. Further details of how the model has been parametrized to biophysical measurements are given in [36], [41].

### Numerical simulations

We use a 400×400 hexagonal grid with periodic (toroidal) boundary conditions and grid mesh size equivalent to 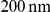. A time step corresponds to 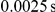, and the same timestep is used to numerically integrate the PDEs. Diffusion processes were integrated using the Alternating Direction Implicit (ADI) method [90], but modified to be performed in units of one-third timestep along each of the three principal directions given by the hexagonal symmetry of the field.

At a retracting site, all filaments and barbed ends that were in that site are pushed back with the edge, and pile up at adjacent sites with their original orientations. Their barbed ends push against the new edge, and some become load-bearing. When the edge protrudes outwards, barbed ends formerly pushing lose contact with the membrane. In this way, filaments and barbed ends are not lost or generated *de novo* when the membrane retracts or extends, and the build-up and release of internal forces are directly coupled to the cytoskeleton. Fig. 9 illustrates this process.

Moreover, as the cell moves (due to the dynamics given by Eqn. 16) the edge of the cell deforms, and hence the local unit normal vector changes, which results in changes of the boundary conditions for which the intracellular dynamics are run (see Eqn. (6)). As explained above, we choose to update concentrations locally in such a way that we preserve mass conservation (nothing is added or lost in a pixel extension or retraction).

## Supporting Information

Video S1 **Rac distributions in migrating** ***in silico*** **cells without (left) and with (right) PI feedback.** Video showing the dynamics of the cells depicted in Fig. 3. Colour map indicating Rac levels is as defined in Fig. 2.
(MPG)

Video S2 **Effect of cell shape and intracellular ‘front-back’ interface curvatures.** Video of the dynamics of the Cdc42 distribution profiles in static, elliptically shaped *in silico* cells without (left) and with (right) PI feedback, as shown in Fig. 4a. Colour map indicates Cdc42 levels as defined in Fig. 2.
(MPG)

Video S3 **Recovery from a disturbance in the ‘front-back’ interface.** Video of the dynamics of the Cdc42 distribution profiles in static *in silico* cells without (left) and with (right) PI feedback. Two distinct types of disturbances are applied, as in Fig. 4b,c. The ‘front-back’ interface rapidly straightens out. Colour map indicates Cdc42 levels as defined in Fig. 2.
(MPG)

Video S4
**Feedback of cell shape dynamics on intracellular dynamics.** A comparison between Cdc42 repolarization in an *in silico* cell whose shape is frozen (left) with a control cell in which small G-protein levels dynamically control cell shape via actin dynamics (right), as shown in Fig. 5. Upper panels show a 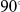 repolarization, while lower panels show a 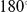 repolarization. The feedback from shape to intracellular dynamics significantly speeds up the reorientation. Colour map indicates Cdc42 levels as defined in Fig. 2.(MPG)

Video S5
**Level of cell shape deformation affects feedback strength to intracellular dynamics.** A comparison between Cdc42 repolarization in an *in silico* cell whose level of shape deformation is more restricted (left, and shown in Fig. 5 and Video S4), and a cell with a more flexible cell shape, due to a threefold reduced coupling energy (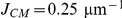) and no membrane stiffness (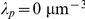). The video shows a 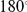 repolarization. Increased cell flexibility significantly speeds up the reorientation even further. Colour map indicates Cdc42 levels as defined in Fig. 2.(MPG)

Video S6 **Effect of phosphoinositide feedback on the response of static cells to conflicting stimuli.** Video of the dynamics of the Cdc42 distribution profiles in static *in silico* cells without (left) and with (right) PI feedback, after simulation with a “V” shaped gradient during 10 s, as shown in Fig. 6a. Colour map indicates Cdc42 levels as defined in Fig. 2.
(MPG)

Video S7 **Effect of phosphoinositide feedback on the response of dynamic cells to conflicting stimuli.** Video of the cell dynamics and Cdc42 distribution profiles in dynamic *in silico* cells without (left) and with (right) PI feedback, after simulation with a “V” shaped gradient during 10 s, as shown in Fig. 6b. Colour map indicates Cdc42 levels as defined in Fig. 2.
(MPG)

Video S8 **Effect of phosphoinositide feedback on the response of dynamic cells to noisy stimuli.** Video of the cell dynamics and Cdc42 distribution profiles in dynamic *in silico* cells without (left) and with (right) PI feedback, after simulation with the same noisy stimulus that is sufficiently large to trigger multiple fronts, as shown in Fig. 6c. Colour map indicates Cdc42 levels as defined in Fig. 2.
(MPG)

Video S9
**Response of cells with varying degrees of PI feedback to a wall.** Cells initially polarized and moving rightwards encounter a wall (green vertical band), as shown in Fig. 7. Video shows the cell dynamics and Cdc42 distribution profiles in *in silico* cells without (left), with normal (middle), and with high (right) PI feedback (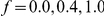, respectively). Colour map indicates Cdc42 levels as defined in Fig. 2.(MPG)

Video S10 **Response of a cell with and without PIs to obstacles in its path.** Video of the cell dynamics and Cdc42 distribution profiles in dynamic *in silico* cells without (left) and with (right) PI feedback. The cell is initially polarized and moving to the right until it encounters the obstacle (static green circular object), as shown in Fig. 8. Colour map indicates Cdc42 levels as defined in Fig. 2.
(MPG)
